# Simultaneous Detoxification of Aflatoxin B1, Zearalenone and Deoxynivalenol by Modified Montmorillonites

**DOI:** 10.3390/molecules27010315

**Published:** 2022-01-05

**Authors:** Jiaqi Mao, Ying Zhou, Guanglie Lv, Renxian Zhou

**Affiliations:** 1 Institute of Catalysis, Zhejiang University, Hangzhou 310028, China; 21737043@zju.edu.cn (J.M.); gllu@zju.edu.cn (G.L.); 2 Zhejiang Ecological and Environmental Monitoring Center, Hangzhou 310012, China; dabaozhou098@163.com

**Keywords:** adsorption, organic-modified montmorillonite, thermal treatment, aflatoxin B1, zearalenone, deoxynivalenol

## Abstract

Raw Ca-based montmorillonite (MMT) was treated by H_2_SO_4_, calcination and organic compounds (hexadecyltrimethyl ammonium bromide (HTAB), cetylpyridinium chloride (CPC) and chitosan (CTS)), respectively. The modified montmorillonites were characterized by different methods and their adsorption performances for three mycotoxins (Aflatoxin B1 (AFB1), zearalenone (ZEA) and deoxynivalenol (DON)) were evaluated at pH = 2.8 and 8.0, respectively. The results indicate that surfactants (CPC and HTAB) intercalation is the most efficient modification, which obviously improves the adsorption performance of montmorillonite for mycotoxins, with adsorption efficiency of above 90% for AFB1 and ZEA whether under acid or alkaline conditions, due to the increase in basal spacing and the improvement of hydrophobicity. Moreover, the adsorption efficiencies of AFB1 and ZEA over CPC-modified montmorillonite (CPC-AMMT-3) coexisting with vitamin B6 or lysine are still at a high level (all above 94%). All modified montmorillonites, however, have low adsorption efficiency for DON, with somewhat spherical molecular geometry.

## 1. Introduction

Contamination of cereal grains and animal feeds with mycotoxins has been widely taken into consideration globally [1]. Mycotoxins are low-molecular-weight secondary metabolites produced by filamentous fungi that have adverse effects on humans and animals under quite low concentration. The liver and kidney are the major target organs for toxicity, but the intestine is also a possible target [2,3]. The economic consequences of mycotoxin contamination are very significant [4]. Therefore, effective removal of mycotoxins which are highly toxic to humans and animals is becoming one of the most urgent challenges.

Presently, the most promising and economical approach to detoxifying mycotoxin-contaminated animal feedstuffs is the addition of nutritionally inert mineral adsorbents to the diet to decrease the bioavailability of mycotoxins in the gastrointestinal tract [5]. Montmorillonite (MMT) has become one of the hotspots in the research field of mycotoxin adsorption due to its excellent adsorption performance, chemical stability and biocompatibility [6]. The active sites of raw MMT with layered structure, however, are usually covered by some impurity [7], which leads to high consumption but low adsorption capacity [8]. Therefore, some methods of modification for raw MMT are studied to improve its adsorption performance [9,10]. For example, the organic modifications and thermal treatment of MMT are two important methods to improve the adsorption performance of raw MMT [11]. Organic-modified MMT refers to MMT modified with long-chain organic cations (surfactants), polymers or polysaccharide molecules, which are generally bonded to MMT by covalent bonds, ion bonds, hydrogen bonds, dipoles or van der Waals forces [12]. Compared with raw MMT, organic-modified MMT has a higher hydrophobicity, which facilitates the adsorption of nonpolar or weak-polar mycotoxins such as zearalenone (ZEA) [13]. Generally, quaternary ammonium salt cationic surfactants such as octadecyltrimethyl ammonium chloride (OTAC), cetyltrimethyl ammonium chloride (CTAC) and hexadecyltrimethyl ammonium bromide (HTAB) are used as the common modifiers to synthesize organic-modified MMT. Feng et al. [13] reported that MMT modified with HTAB (named as CMN) showed great adsorption capacity for ZEA (8.83 mg/g), much higher than raw MMT (0.60 mg/g), and the desorption efficiency of ZEA for MMT and CMN was 22% and 5.8%, respectively. Except for cationic surfactants, polysaccharides have recently been used as modifiers for MMT [14]. Under weak-acid condition, the amide groups are protonated and intercalated into layers of MMT by way of ionic exchange. The composites made of MMT and polysaccharides take advantage of thermal stability and versatility of inorganic and organic materials [15], which have been widely applied in membranes, sensors and pollutant adsorbents, but rarely as mycotoxin binders. Moreover, thermal treatment is also an important method to activate MMT [16], which can lead to the desorption of free water, interlayer water and loss of OH units [17]. After thermal treatment, the adsorptive resistance of water film to organic matter will be reduced, which is conducive to mycotoxin adsorption [18].

Recently, some studies have indicated that the layers of raw MMT could be deeply exfoliated by acid treatment, which is an essential means to change its physicochemical property [10,19]. The acid-modified montmorillonites possess more active sites at the terminal hydroxyl surface, a larger specific area and higher pore volume than raw MMT. Meanwhile, the reactivity of MMT without permission by depth-delaminating treatment is lower, and is also to the disadvantage of organic macromolecular intercalation modification. For example, our research verified that the layers of raw MMT would be delaminated to 2–3 layers after acid treatment under H_2_SO_4_/raw MMT mass ratio of 0.3 and high temperature conditions, not only resulting in the impurity of raw MMT being removed, but also more active sites being exposed. Therefore, the adsorption efficiency of aflatoxin B1 (AFB1) in alkaline conditions is obviously improved over the modified MMT, increasing from 29.3% to 97.7% at pH = 8.0 [10].

Based on the above, this work further evaluated the adsorption capacity of the montmorillonite modified with different methods in vitro. Natural Ca-based montmorillonite was treated by a small amount of H_2_SO_4_ at high temperature, and further modified by calcination, hexadecyltrimethyl ammonium bromide (HTAB), cetylpyridinium chloride (CPC) and chitosan (CTS), respectively. The physicochemical properties of the modified montmorillonites were determined by various techniques, such as X-ray powder diffraction (XRD), N_2_ adsorption–desorption, elemental analysis, in situ infrared Fourier transform (in situ FTIR) and temperature-programmed desorption (TPD) of acetone or benzene. Furthermore, their adsorption performances in a ternary contaminant system of three mycotoxins (AFB1, ZEA and DON) were evaluated at pH = 2.8 and 8.0 in order to simulate the conditions of the stomach and small intestine, respectively. Moreover, the selective adsorption efficiency of mycotoxins over the CPC-modified montmorillonite present in vitamin B6 or lysine has also been discussed. The purpose is to gain some new insights into the relationship between the adsorption performances and structure of modified montmorillonites.

## 2. Materials and Methods

### 2.1. Materials

Natural Ca-based montmorillonite (denoted as MMT) with purity higher than 75% was obtained from China. The hexadecyltrimethyl ammonium bromide (HTAB), cetylpyridinium chloride (CPC) and chitosan (CTS) used for the preparation of intercalated MMT were supplied by Sinopharm Chemical Reagent Co. Ltd. (Shanghai, China) The degree of deacetylation of chitosan was at 90%. The mycotoxins such as AFB1, DON and ZEA were purchased from J&K Chemical Co. (Beijing, China) Primary stock solutions of each mycotoxin (100 μg/mL) were prepared in chromatographic-grade methanol and stored at 2 °C.

### 2.2. Acid Treatment

5 g MMT powder was mixed with 100 mL aqueous solution of sulfuric acid, then placed in a three-necked flask at 90 °C for 3 h. In this reaction system, the 98% H_2_SO_4_/MMT ratio (*w*/*w*) was 0.3. The resulting acid-activated MMT was separated by centrifugation (4440 g) and repeatedly washed with de-ionized water until excess SO_4_^2−^ ions were not detected by the barium nitrate test. The obtained sample was dried at 60 °C overnight and ground to a size less than 75 μm. The sample was denoted as AMMT-3.

### 2.3. Intercalated Montmorillonite Preparation

The HTAB- and CPC-intercalated AMMT-3 samples were prepared via an ion exchange reaction: 2 g AMMT-3 was dispersed in 50 mL distilled water for 30 min, and then 1 g HTAB or CPC added into the mixture. The mixture was stirred overnight at room temperature, then separated by centrifugation (4440 g). The solid product was washed with de-ionized water (until Br^−^ or Cl^−^ were not detected by the silver nitrate test) followed by drying at 60 °C. The samples were denoted as HTAB-AMMT-3 and CPC-AMMT-3, respectively.

The CTS-intercalated AMMT-3 was also prepared via an ion exchange method described by Topcu et al. [20]. 1 g chitosan was slowly added to 100 mL 3% acetic acid aqueous and stirred under 70 °C to protonate amino groups. 2 g AMMT-3 was then added into the solution above, and the mixture was continuously stirred for 6 h, followed by centrifugation (4440 g). The solid product was washed with distilled water and 1% acetic acid aqueous twice, respectively, to remove excess chitosan. The sample was dried at 60 °C and denoted as CTS-AMMT-3.

### 2.4. Thermal Treatment

The AMMT-3 was treated in a quartz tube reactor at 350 °C for 3 h in an argon atmosphere (30 mL/min) to remove the interlayer coordinated water of montmorillonite. The thermal treated sample was denoted as 350-AMMT-3.

### 2.5. Mycotoxin Adsorption Test

AMMT-3, HTAB-AMMT-3, CPC-AMMT-3, CTS-AMMT-3 and 350-AMMT-3 were selected to perform in vitro mycotoxin adsorption by the method described in previous literature [21]. The mixed solution containing AFB1, ZEA and DON (200 ng/mL each) was obtained by diluting the primary stock solutions of mycotoxins. For simulating the conditions of a poultry stomach and small intestine, phosphate-buffered saline (PBS) was taken to adjust the pH of mixed solution to 2.8 and 8.0, respectively. A conical tube was then filled with 5 mL of the mycotoxin mixed solution above and 20 mg of each solid sample. The tubes were shaken in an incubator at 37 °C for 4 h followed by centrifugation. Next, 4 mL methyl tert-butyl ether (t-BME) was used to extract mycotoxins from 4 mL supernatant. Finally, the dry remainder after solvent evaporation was redissolved in 200 μL acetonitrile to detect the residual unbound AFB1, ZEA and DON concentration by LC-MS/MS analysis.

The High-Performance Liquid Chromatography (HPLC) system consisted of Agilent (Santa Clara, CA, USA) 1290 pump and autosampler system with a Zorbax SB (Santa Clara, CA, USA) C-18 HPLC column (2.1 mm × 150 mm; i.d. 3.5 μm) and a pre-column of the same type. The injection volume was 5 μL. The mobile phases were HPLC-grade water (A) and acetonitrile (B) and the flow rate was set at 0.3 mL/min (see Table 1). The MS/MS detection system was Agilent 6460 (Santa Clara, CA, USA), operated in the ESI-negative mode. The *m*/*z* transitions for quantification were 313.2 > 285.1 (AFB1), 319 > 301.2 (ZEA) and 297 > 279.1 (DON). The capillary and cone voltages were 3 kV and 500 V, respectively, and source temperature was set at 325 °C and sheath gas temperature at 350 °C. The cone gas flow and sheath gas flow were set at 5 L/min and 11 L/min, respectively, and the optimized collision energy was 22 eV (AFB1) and 4 eV (ZEA/DON). Calibration curves were attained using five standards over the concentration range of the sorption samples and repeated three times. Chromatograms and mass spectra are shown in Figures S2 and S3. The (%) of AFB1, ZEA and DON were calculated as follows:

$$\mathrm{Adsorption}\;\mathrm{efficiency}=\frac{C_{0}{-C}_{1}}{C_{0}}\times 100\%$$

where C_0_ and C_1_ are the equilibrium concentration of control groups and experimental groups.

### 2.6. Adsorption Test at Different Montmorillonite/Mycotoxin Mass Ratio

CPC-AMMT-3 was selected as the mycotoxin adsorbent to further study the adsorption capacity of modified adsorbents by altering montmorillonite/mycotoxin mass ratio. Two conical tubes were both filled with 5 mL buffer solution of mycotoxins and 10 mg or 5 mg CPC-AMMT-3, respectively. Experiments were conducted according to the same procedure described in Section 2.5.

### 2.7. Mycotoxin/Nutrient Selective Adsorption Test

CPC-AMMT-3 was also selected as the mycotoxin adsorbent to study adsorption performance of modified adsorbents in presence of nutrients such as vitamin B6 or lysine. 4.8 mg vitamin B6 and 3.4g lysine, each in duplicate, were added in four 250 mL brown volumetric flasks and diluted with buffer solution to volume by the aqueous solution of mycotoxins with a pH of 2.8 and 8.0, respectively. A conical tube was filled with 5 mL aqueous solution of mycotoxins/nutrient and 20 mg CPC-AMMT-3. The tubes were shaken in an incubator at 37 °C for 4 h followed by centrifugation. The following experimental procedure is the same as 2.5.

### 2.8. Characterization

The total cation exchange capacity (CEC) of all samples was tested by hexamminecobalt trichloride solution–spectrophotometric method [20]. Phase composition of samples was analyzed using XRD on a Bruker Advance 8 (Karlsruhe, Germany) diffractometer. Operating parameters were as follows: Cu-Kα radiation, 40 kV and 40 mA, in the 2θ range of 2–40° with a scanning rate of 5°/min and step size of 0.02°.

The N_2_ adsorption–desorption was measured on a Micromeritics TriStar II instrument (Atlanta, GA, USA). Before the measurement, the samples were degassed under vacuum at 120 °C for 6 h. The surface area and the distribution of pore sizes of the samples were calculated by the Brunauer–Emmett–Teller (BET) method and Barrett–Joyner–Halenda (BJH) method, respectively.

The contents of carbon, nitrogen and hydrogen in the intercalated montmorillonites were determined by Vario Micro elemental analyzer (Elementar, Hanau, Germany). These data were used to estimate the intercalated amounts of HTAB, CPC and CTS.

In situ diffuse reflectance infrared Fourier transform spectra (Nicolet 6700 instrument equipped with an MCT detector) (Thermo Fisher Scientific, Waltham, MA, USA) were obtained to characterize the molecular structure of modified montmorillonite. 100 mg of montmorillonite was purged with Ar (90 mL/min) at 60 °C for 30 min to remove the contaminant, then infrared spectrum data started to be collected. The scanning range was 4000–1000 cm^−1^, and the spectral resolution was 4 cm^−1^. After deducting the Ar background from the collected raw data, the infrared spectrum of the sample was obtained.

Temperature-programmed desorption (TPD) of acetone or benzene was measured in a quartz fixed-bed micro-reactor (Micromeritics, Atlanta, GA, USA) equipped with TCD detector. Firstly, the sample (100 mg, 40~60 meshes) was pretreated in N_2_ stream at 60 °C for 0.5 h. After being cooled to 35 °C, the sample was exposed to a flow of acetone or benzene vapor mixed with N_2_ (30 mL/min) until adsorption equilibrium was achieved. The sample was then treated in N_2_ (30 mL/min) for 0.5 h to remove physically absorbed acetone or benzene. Finally, desorption experiment was conducted from 35 to 400 °C at a heating rate of 10 °C/min.

## 3. Results and Discussion

### 3.1. Structure Analysis

**Figure 1:**
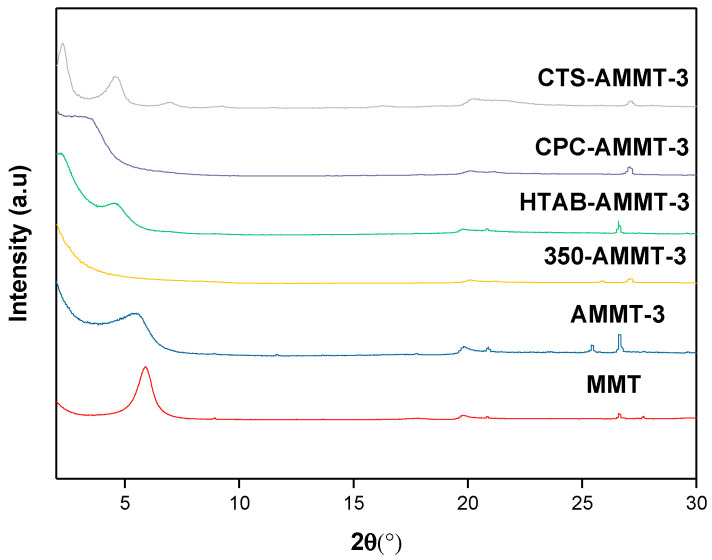
XRD profiles of raw MMT and modified montmorillonites.

The XRD patterns and corresponding data of raw MMT and modified MMT samples are shown in Figure 1 and Table 2, respectively. According to the previous research [10], we verified that the broadening {001} reflection peak of AMMT-3 may suggest that montmorillonite layers were exfoliated during the acid activation process at a high temperature. Moreover, the XRD pattern of the thermal treated sample 350-AMMT-3 shows that diffraction intensity and basal spacing (d_001_) of {001} reflection decreases sharply compared with AMMT-3 (d_001_ = 15.9 Å), which indicates a great influence of thermal modification on the crystal structure of montmorillonite. The reduction of d_001_ is generally attributed to the loss of physically absorbed and interlayer coordinated water [17]. On the other hand, the disappearance of the 001 reflection also indicates the destruction of the part-crystal structure to a certain extent.

With regard to the CPC-AMMT-3 and HTAB-AMMT-3 samples intercalated by surfactants CPC and HTAB, the d_001_ is about 25.8 Å and 39.0 Å, respectively, which shows the successful intercalation of alkyl chains. According to the literature [22], the thickness of a single dioctahedral platelet is about 9.7 Å. The molecular length of CPC and HTAB is 23.1 Å and 25.3 Å, respectively. In the previous study [10], we proved a paraffin-type bimolecular arrangement of HTAB, and the tilting angle of the alkyl chains was 35.1°. Similarly, it can be also calculated that CPC has a paraffin-type single molecular arrangement and the tilting angle is 43°. For CTS-AMMT-3 sample intercalated by CTS, the appearance of its second and third order reflection {002} and {003} proves good crystallization. Unlike the chain configuration of CPC and HTAB, CTS is made up of space grid structure. The interlayer distance of CTS-AMMT-3 is 38.4 Å, which is about several times higher than the width of chitosan (3.7~4.3 Å) and may imply an unordered multilayer arrangement. The chitosan molecules are firmly immobilized between the layers because of electrostatic attraction between protonated amide groups and layers of montmorillonite with negative charges.

### 3.2. Elemental Analysis

Elemental analysis of N was performed on organic-modified AMMT-3 to evaluate the intercalation loadings. As listed in Table 3, the CTS load in CTS-AMMT-3 is 1.24 mmol/g, which is the highest among three organic-modified montmorillonites, and much larger than CEC value of AMMT-3 (0.57 mmol/g). It can be inferred that one part of the chitosan intercalated is bound to the negatively charged surface of layers by electrostatic attraction, while the other part is fixed between layers by intermolecular hydrogen bonding. Gunister et al. [23] proposed a molecular model of chitosan/montmorillonite composite based on the research results of rheology and electrokinetics. Polysaccharide molecules can not only be adsorbed on the surface of lamellas, but also cross-linked with each other to reduce the dispersion of clay in solution.

Additionally, the HTAB load in the HTAB-AMMT-3 is lower than that of CPC-AMMT-3 and approximately equal to the initial concentration of intercalation agent. As mentioned in the results of XRD above, the arrangements of CPC and HTAB are single and bimolecular paraffin type, respectively. It can be speculated that the pyridine ring with lower steric hindrance of CPC benefits a denser arrangement, which leads to the higher load amount. The existence of organic compounds in the organic-modified AMMT-3 samples was also verified by FTIR. As shown in Figure S1, compared to MMT and AMMT-3, many new absorption peaks related with CPC in CPC-AMMT-3 can be observed. The absorption peak at 1465 cm^−1^ can be attributed to the bending vibration of -CH_2_ in the alkyl chain, and its symmetrical and asymmetrical stretching vibration peaks are located at 2850 cm^−1^ and 2928 cm^−1^, respectively. The peak at 1638 cm^−1^ is formed by the C=C and C=N stretching vibrations on the pyridine ring. Moreover, the intensity of -OH peaks in the CPC-AMMT-3 at 1620 cm^−1^ and 3420 cm^−1^ was obviously weakened, which means that the interlayer water in the CPC-modified montmorillonite is greatly reduced, resulting in its hydrophobicity being enhanced.

### 3.3. Texture Analysis

**Figure 2:**
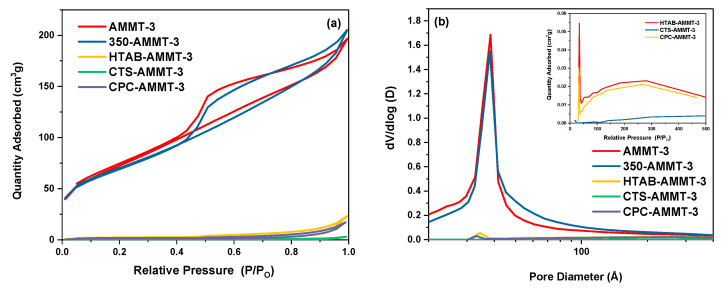
N_2_ adsorption–desorption isotherms (**a**) and pore size distribution (**b**) of the modified montmorillonites.

The N_2_ adsorption–desorption isotherms and pore size distribution of the modified samples are shown in Figure 2. As presented in Figure 2a, the isotherm and hysteresis loop of 350-AMMT-3 is of type IV and H_4_, respectively, which is both in good agreement with AMMT-3 and typical for mesoporous materials. It is concluded that the layer structure of 350-AMMT-3 is retained while interlayer coordinated water was lost after calcination. The H_4_ hysteresis loop is formed by slit-shaped pores resulting from multilayer formation.

As shown in Figure 2a and Table 4, the specific surface area and pore volume of HTAB-AMMT-3, CTS-AMMT-3 and CPC-AMMT-3 all are greatly reduced even if the type of isotherms and hysteresis loops remain unchanged compared with AMMT-3. This may be due to alkyl or polysaccharide chains blocking the N_2_ sorption sites in the layers and pore network. It is worth noting that for the surfactants (CPC and HTAB)-modified montmorillonites, the specific surface area and pore volume of CPC-AMMT-3 are less than those of HTAB-AMMT-3, because the lower steric hinderance of pyridine ring compared with alkyl chains facilitates a denser arrangement of CPC in the interlayer and more occupancy of N_2_ sorption sites. Furthermore, the specific surface area and pore volume of CTS-AMMT-3 are smallest among all samples, which is related with blockages of layers and mesopores by large molecules. This is in agreement with the experiment result from XRD and elemental analysis.

As presented in Figure 2b, pore size distributions of AMMT-3 and 350-AMMT-3 are concentrated and the average pore size is 46 Å and 51 Å, respectively. A large number of mesopores are formed by the interlamellar region and edge/face contacts of exfoliated layers of montmorillonites. In addition, pore sizes of HTAB-AMMT-3 and CPC-AMMT-3 gather between 40 Å and 50 Å, while those of CTS-AMMT-3 range from 100 Å to 500 Å. These results suggest that for organic-modified AMMT-3, the surfactant molecules (CPC and HTAB) are limited in the interlayer while chitosan molecules are evenly distributed on the inner and outer surfaces of layers. Therefore, the CTS load amount in CTS-AMMT-3 is the highest among the three organic-modified montmorillonites.

### 3.4. Acetone/Benzene-TPD

**Figure 3:**
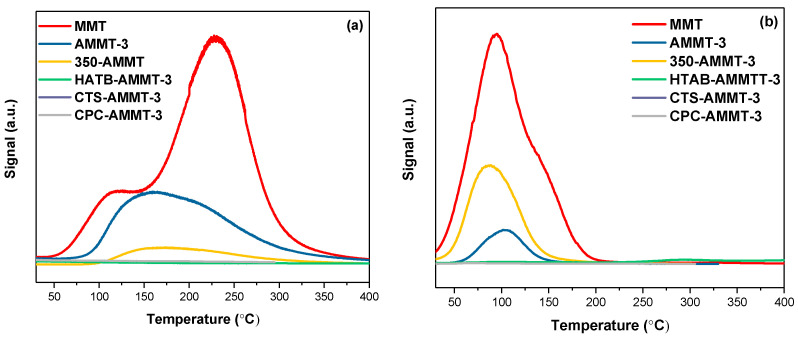
Profiles of acetone-TPD (**a**) and benzene-TPD (**b**) of raw MMT and modified montmorillonites.

Acetone/benzene-TPD were carried out to understand the adsorption performance of the organic-modified samples towards the organic compounds with different polarity and the properties of surface adsorption sites, since mycotoxins are organic compounds with different polarity and functional groups. As shown in the profiles of acetone-TPD in Figure 3a, both AMMT-3 and 350-AMMT-3 exhibit two overlapping peaks in the temperature range of 100~400 °C, which is related to the desorption of acetone interacting with the negatively charged montmorillonite surface and exchangeable-cation hydration-shell water. However, there is only an acetone desorption peak with a small area over HTAB-AMMT-3, CTS-AMMT-3 and CPC-AMMT-3, which can be ascribed to the blockages of layers and mesopores by organic molecules reducing adsorption sites. This is consistent with the result of N_2_ adsorption–desorption mentioned above. As the profiles of benzene-TPD in Figure 3b, all of the samples have only one desorption peak in the range from 30 to 150 °C, suggesting that the electron-rich and nonpolar benzene molecules only interact with interlayer cation rather than layers. This may explain the reason why the peak area of 350-AMMT-3 is larger than that of AMMT-3. As discussed in XRD results, 350-AMMT-3 lost interlayer coordinated water due to thermal modification, which is favorable for the adsorption of more benzene.

### 3.5. Adsorption Performance of Mycotoxins

#### 3.5.1. Adsorption Performance of Modified Montmorillonites for Mycotoxins

**Figure 4:**
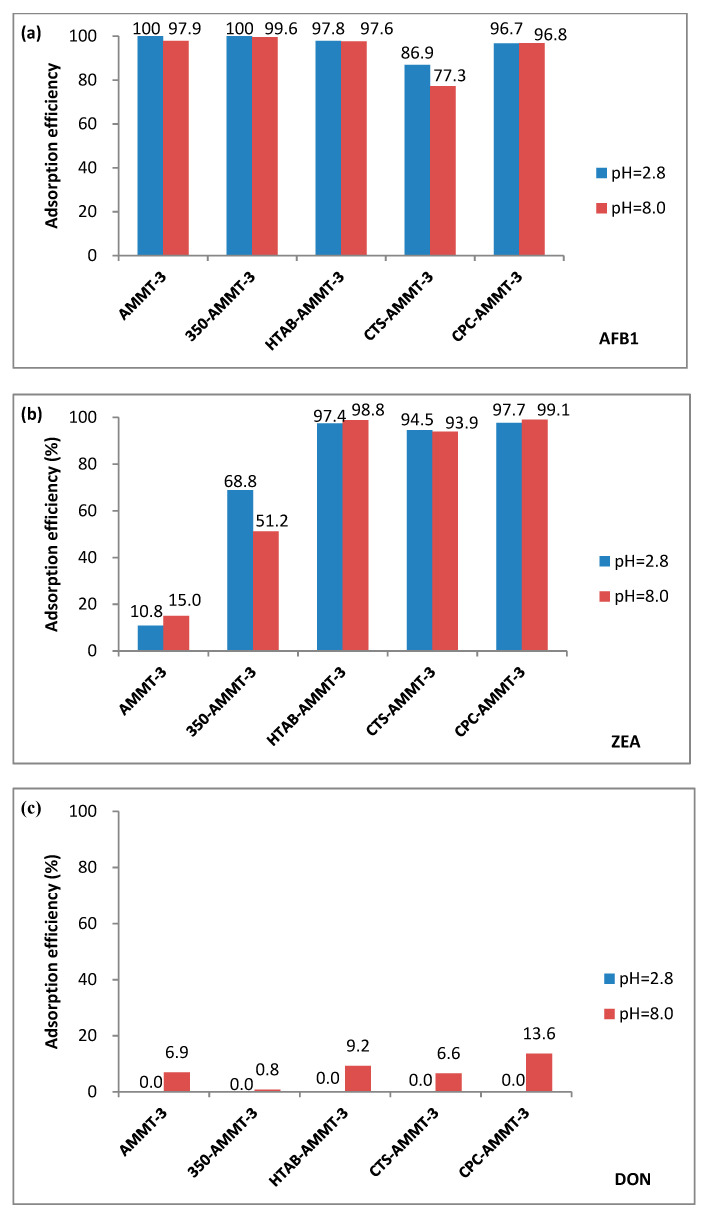
Adsorption efficiency of AFB1 (**a**), ZEA (**b**) and DON (**c**) over the modified montmorillonites.

**Figure 5:**
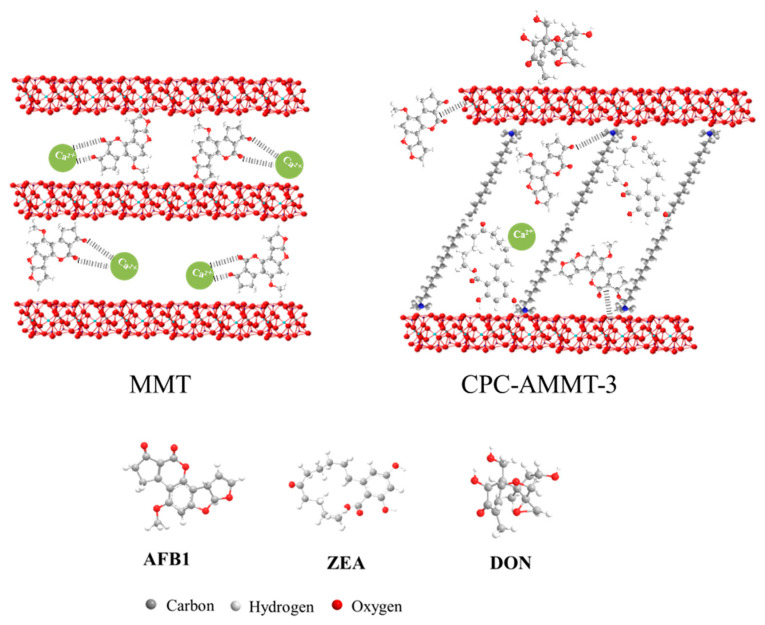
Schematic mechanism of AFB1, ZEA and DON adsorption on MMT and CPC-AMMT-3.

Figure 4 shows the adsorption efficiency of the modified montmorillonites to AFB1, ZEA and DON under acid or alkaline conditions, respectively, and the schematic mechanism of AFB1, ZEA and DON adsorption on MMT and CPC-AMMT-3 is displayed in Figure 5. As displayed in Figure 4a, the adsorption efficiency of the samples for AFB1 is over 96.0% except for CTS-AMMT-3, and CTS-AMMT-3 only obtains the adsorption efficiency of 86.9% and 77.3% under acid and alkaline conditions, respectively. Based on our previous work [10], there are three dominating adsorption sites on montmorillonite, including edges, external surface and interlayer space. The layers of raw MMT are exfoliated after acid treatment, exposing more active sites at the terminal and external surface for the adsorption of AFB1 (illustrated in Figure 5). According to the characterization of CTS-AMMT-3 mentioned above, chitosan molecules distribute on the inner and outer surfaces of layers and cover some adsorption sites of AFB1. This adsorption site cover not only hinders the formation of hexatomic rings by chelating the β-dicarbonyl system of AFB1 with metal ions in the interlayers or edges, but also hinders the attraction of the negatively charged montmorillonite surface to the partial positively charged carbon atoms of β-dicarbonyl groups.

Comparing the adsorption efficiency of ZEA over AMMT-3 and 350-AMMT-3, the former is only 10.8% and 15.0%, while the latter is 68.8% and 51.2% under acid and alkaline conditions, respectively. The result indicates that the hydration-shell water exhibiting before thermal modification prevents the adsorption of ZEA. As for organic-modified AMMT-3, the adsorption efficiency for ZEA is over 90% whether under acid or alkaline conditions, far higher than those of AMMT-3. However, Wang et al.’s research indicated that the montmorillonites modified by surfactant only showed super-enhanced adsorption rates towards ZEA under acidic conditions but poor performance under alkaline conditions [24]. As discussed above, the increase in basal spacing and the improvement of hydrophobicity could enhance the adsorbability of ZEA over montmorillonite (illustrated in Figure 5). Thus, the organic modification on the surface of AMMT-3 activated by inorganic acids is the most efficient among the modified methods.

Figure 4c exhibits the adsorption efficiency of the modified samples for DON. The adsorption efficiency of all samples for DON are very low, whether in acid or alkaline conditions (no more than 15%), which agrees with the conclusion reported in previous literature [25]. It could be identified that the poor planarity of DON leads to the largest steric hindrance, preventing it from entering into the interfacial layer of montmorillonite and interacting with intercalated organic molecules (illustrated in Figure 5).

#### 3.5.2. Effect of Different Montmorillonite/Mycotoxin Mass Ratios on Adsorption Performance of CPC-AMMT-3

**Figure 6:**
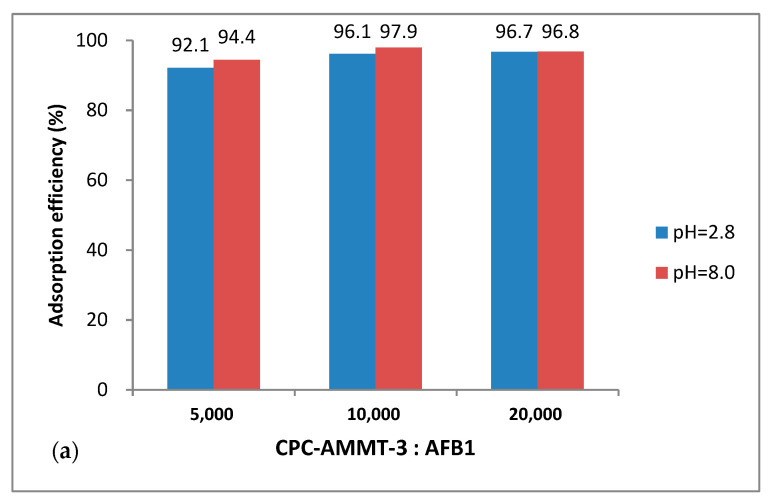

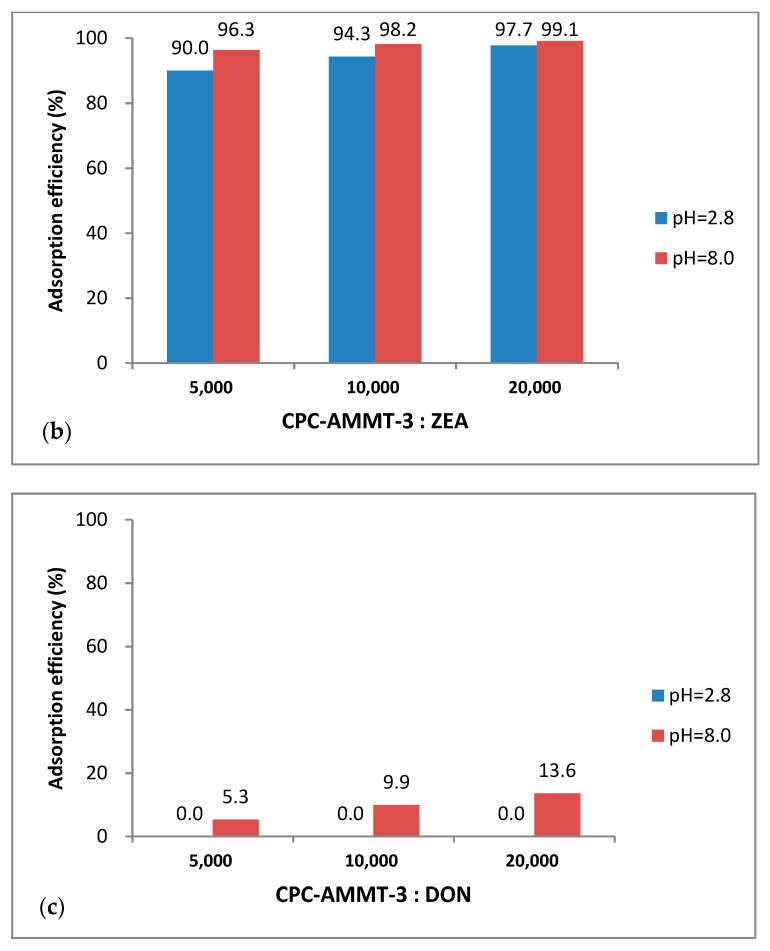
Adsorption efficiency of AFB1 (**a**), ZEA (**b**) and DON (**c**) with different montmorillonite/mycotoxin mass ratios.

As discussed above, CPC-AMMT-3 exhibits the best adsorption performance among all modified samples, whether under acid or alkaline conditions. Thus, the sample was selected to further study the adsorption capacity of modified montmorillonites for each mycotoxin by altering montmorillonite/mycotoxin mass ratios, and the results are shown in Figure 6. It can be seen from Figure 6 that when the mass ratio of CPC-AMMT-3 with mycotoxin reduces from 20,000 to 10,000, the adsorption efficiency for AFB1 remains unchanged while the adsorption efficiency for ZEA and DON decreases slightly. In addition, when the mass ratio reaches 5000, the adsorption efficiency of CPC-AMMT-3 for AFB1 and ZEA is still over 90%, which shows that CPC-AMMT-3 possesses a higher adsorption capacity for some mycotoxins, as well as excellent application prospects and economical efficiency.

#### 3.5.3. Selective Adsorption Performance of AMMT-3 and CPC-AMMT-3 in Presence of Nutrients

**Figure 7:**
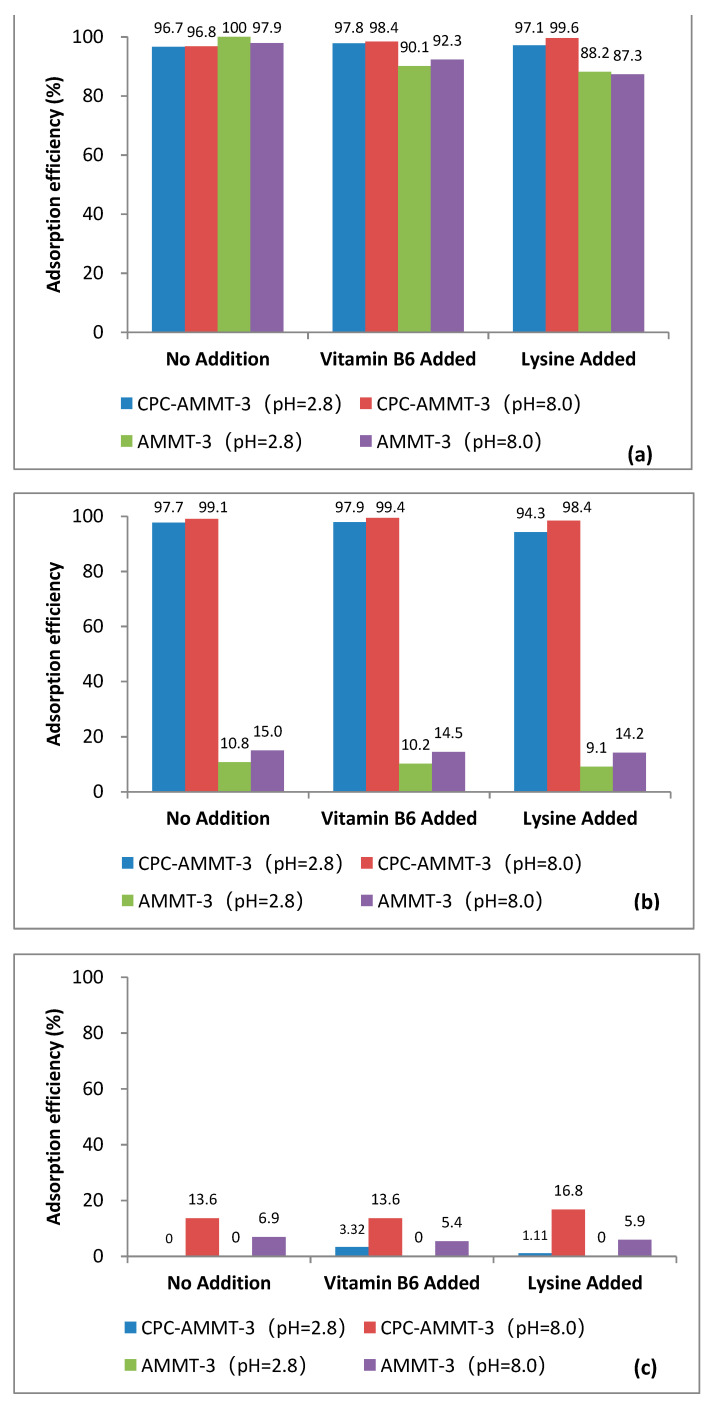
Adsorption efficiency of AFB1 (**a**), ZEA (**b**) and DON (**c**) over AMMT-3 and CPC-AMMT-3 under the condition of vitamin B6 or lysine coexistence.

In practical application, nutrients (such as vitamins and amino acids) in feed may interfere with the absorption of mycotoxin over montmorillonite. The application is of little value if CPC-AMMT-3 also adsorbs nutrients besides mycotoxins. Thus, the adsorption efficiency of mycotoxins over AMMT-3 and CPC-AMMT-3 in the presence of vitamin B6 or lysine has also been evaluated in this work and the adsorption efficiency is shown in Figure 7. As illustrated in Figure 7a,b, the adsorption efficiency of AFB1 and ZEA over CPC-AMMT-3 still maintains over 94% even if vitamin B6 or lysine coexists. However, the adsorption efficiency of AFB1 over AMMT-3 shows significant decline due to vitamin B6 and lysine chelate metal ions within the interlayers or edges to form configuration of hexatomic rings, competing for the same adsorption sites of AFB1. The result suggests that CPC-AMMT-3 exhibits a good selective adsorption performance for detoxifying AFB1 and ZEA, which is not affected by nutrients in application. It can be seen from Figure 7c that under the condition of vitamin B6 and lysine coexistence, the adsorption efficiency of CPC-AMMT-3 for DON is still relatively low.

## 4. Conclusions

A series of modified montmorillonites by H_2_SO_4_, calcination and organic compounds [hexadecyltrimethyl ammonium bromide (HTAB), cetylpyridinium chloride (CPC) and chitosan (CTS)] were prepared and characterized by various techniques such as XRD; N_2_ adsorption-desorption; acetone/benzene-TPD and FTIR; and their adsorption performances for three mycotoxins (AFB1, ZEA and DON) were evaluated at pH 2.8 and 8.0, respectively. The results show that the CPC/HTAB modified montmorillonites (HTAB-AMMT-3 and CPC-AMMT-3) display outstanding adsorption performance for AFB1 and ZEA whether in acidic or alkaline conditions, with adsorption efficiency of above 90% for AFB1 and ZEA. The layers of raw montmorillonite were exfoliated after acid treatment, exposing more active sites at the terminal and external surface for the adsorption of mycotoxins. The intercalation of HTAB and CPC results in the increase in basal spacing and the improvement of hydrophobicity, which enhances the adsorption efficiency for mycotoxins. However, all modified montmorillonites have low adsorption efficiency for DON with somewhat spherical molecular geometry. Moreover, the CPC-modified montmorillonite (CPC-AMMT-3) shows excellent application prospects and economical efficiency. The selective adsorption efficiency of AFB1 and ZEA over CPC-AMMT-3 coexisting with vitamin B6 or lysine is still a high level (all above 94%), and has great adsorption capacity of AFB1 and ZEA, with an adsorption efficiency still above 90% when the mass ratio of CPC-AMMT-3 with mycotoxin reaches 5000.

## Figures and Tables

**Table 1:** Gradient elution procedures of HPLC.

Elution Time (min)	HPLC-Grade Water (%)	Acetonitrile (%)
0.00	90	10
5.00	15	85
8.00	5	95
10.00	5	95
10.50	65	35
13.50	65	35

**Table 2:** XRD data and CEC values of raw MMT and modified montmorillonites.

Samples	2θ (°)	d_001_ (Å)	CEC (mmol/g)
MMT	5.92	14.91	1.06
AMMT-3	5.55	15.9	0.57
350-AMMT-3	8.73	10.1	0.38
HTAB-AMMT-3	2.23	39.0	0
CTS-AMMT-3	2.29	38.4	0
CPC-AMMT-3	3.42	25.8	0

**Table 3:** Elemental analysis data of organo-modified montmorillonites.

Samples	Initial Concentration of Intercalation ^1^ (CEC)	N% ^2^	Load Amount ^3^ (mmol/g)
HTAB-AMMT-3	2.40	1.15	0.82
CTS-AMMT-3	3.16	1.73	1.24
CPC-AMMT-3	2.45	1.28	0.91

^1^ Initial concentration of intercalation = $\frac{m_{A}}{2\cdot M_{A}\cdot {\mathrm{CEC}}_{\mathrm{AMMT}\text{-}3}}$, where m_A_ (1 g) is the initial mass of HTAB, CPC and chitosan, M_A_ is the molecular mass of HTAB, CPC and chitosan (using its monomer glucosamine as the calculating unit), CEC_AMMT-3_ is the total cation exchange capacity of AMMT-3 (0.57 mmol/g). ^2^ N% of samples have been corrected by the background correction methods with unintercalated montmorillonites AMMT-3. ^3^ Load amount is calculated from N analysis.

**Table 4:** Texture properties of the modified montmorillonites.

Samples	S_BET_ (m^2^/g)	V (cm^3^/g)	Pore Size (Å)
AMMT-3	267.0	0.30	46
350-AMMT-3	251.0	0.32	51
HTAB-AMMT-3	7.4	0.04	195
CTS-AMMT-3	1.4	0.01	130
CPC-AMMT-3	4.8	0.03	222

## Data Availability

The data presented in this study are available on request from the corresponding author. The data are not publicly available due to ownership issues.

## Supplementary Materials

The following are available online, Figure S1: DRFTIR spectra of MMT, AMMT-3 and CPC-AMMT-3., Figure S2: Sample Chromatogram. Figure S3: Mass spectras of DON (a), AFB1 (b) and ZEA (c).

## Sample Availability

Samples of the compounds prepared in this article are available from the authors.
