# Supplementary material for: Simultaneous Detoxification of Aflatoxin B1, Zearalenone and Deoxynivalenol by Modified Montmorillonites
Source: Molecules. 2022 Jan 5;27(1):315. doi: 10.3390/molecules27010315 (PMC8746407; doi:10.3390/molecules27010315)
Supplement: Supplementary file 1 [file molecules-27-00315-s001.zip › molecules-1506811-supplementary.pdf]

Supplementary material

Simultaneous Detoxification of Aflatoxin B1, Zearalenone and Deoxynivalenol by Modified Montmorillonites

Jiaqi Mao [1,†], Ying Zhou [2,†], Guanglie Lv [1] and Renxian Zhou [1,*]

[1] Institute of Catalysis, Zhejiang University, Hangzhou 310028, China; 21737043@zju.edu.cn (J.M.); gllu@zju.edu.cn (G.L.)
[2] Zhejiang Ecological and Environmental Monitoring Center, Hangzhou 310012, China; dabaozhou098@163.com
* Correspondence: zhourenxian@zju.edu.cn; Tel.: +86-571-88273290
† These authors contributed equally to this work.

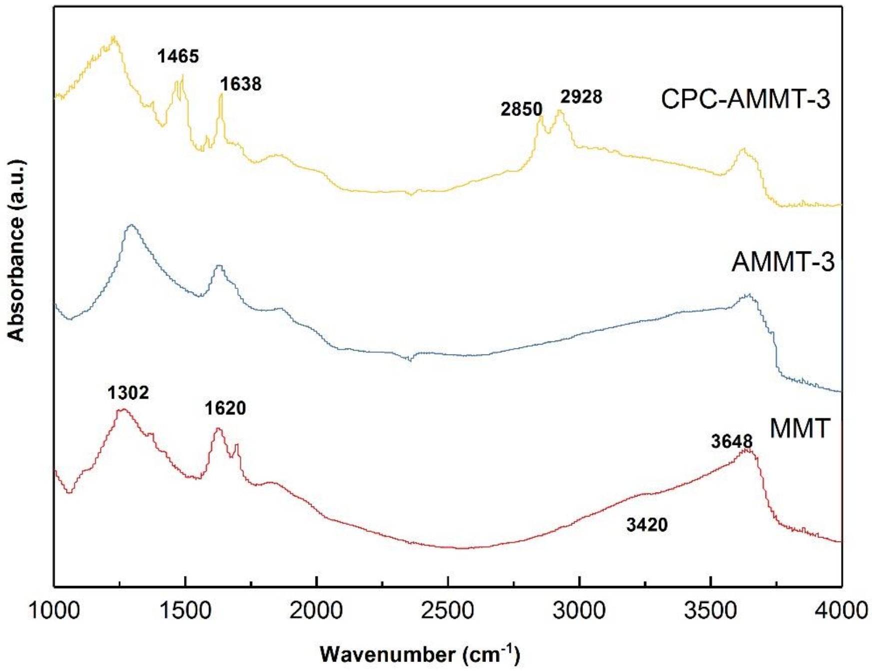

Figure S1. DRFTIR spectra of MMT, AMMT-3 and CPC-AMMT-3.

Figure S1 are the diffuse reflectance infrared spectra of MMT, AMMT-3 and CPC-AMMT-3 in the wavenumber range of 4000-1000 cm^{-1}. It can be seen from **the** Figure S1 that the strong absorption peak at 1302 cm^{-1} in the infrared spectra of MMT and AMMT-3 samples is attributed to the stretching vibration of montmorillonite Si-O, including two forms of in-plane and out-of-plane

vibration. The absorption peak at 1620 cm^{-1} is attributed to the -OH bending vibration peak of the interlayer water of montmorillonite, while the stretching vibration peak appears at 3420 cm^{-1}. The absorption peak at 3648 cm^{-1} is produced by the -OH stretching vibration connected to aluminum in the octahedron. By comparing MMT and AMMT-3, it can be seen that the intensity of -OH peaks in the CPC-AMMT-3 at 1620 cm^{-1} and 3420 cm^{-1} was obviously weakened, which means that the interlayer water in the organic-modified montmorillonite is greatly reduced. Moreover, many new absorption peaks related with CPC in CPC-AMMT-3 can be observed. The absorption peak at 1465 cm^{-1} can be attributed to the bending vibration of $-CH_2$ in the alkyl chain, and its symmetrical and asymmetrical stretching vibration peaks are located at 2850 cm^{-1} and 2928 cm^{-1}, respectively. The peak at 1638 cm^{-1} is formed by the C=C and C=N stretching vibrations on the pyridine ring. The result indicates that AMMT-3 was modified by CPC, resulting in the hydrophobicity being obviously enhanced.

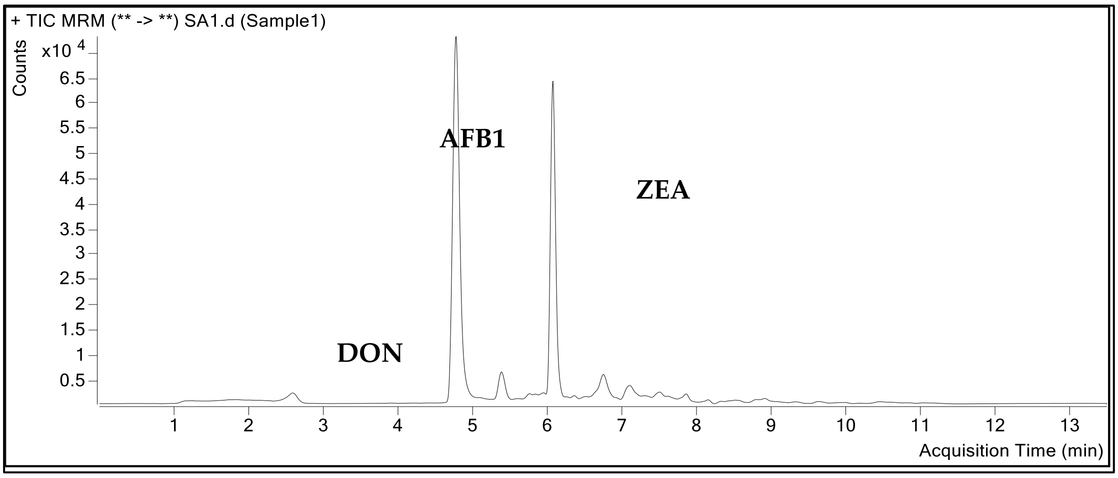

Figure S2. Sample Chromatogram.

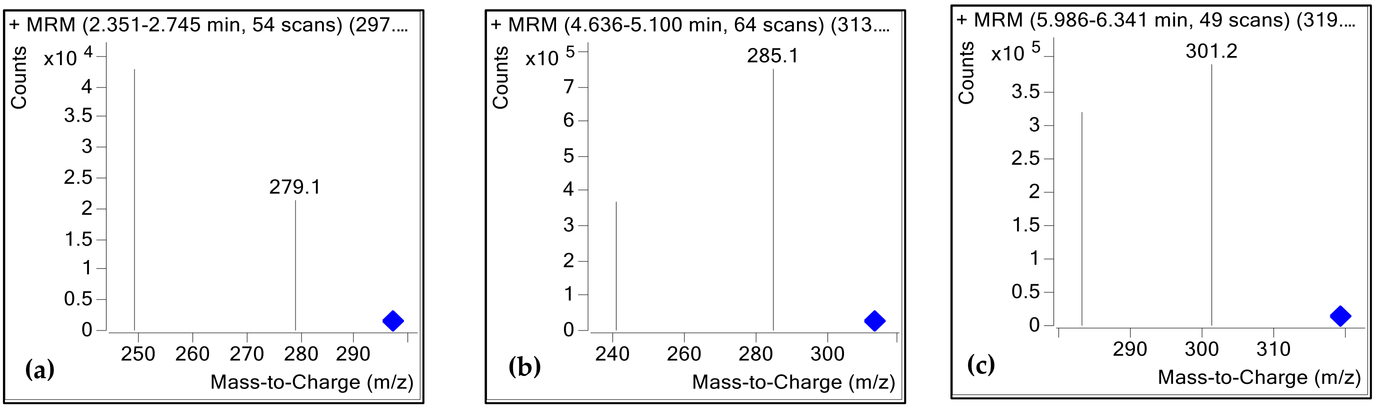

Figure S3. Mass spectras of DON (a), AFB1 (b) and ZEA (c).
